# Supplementary material for: The DBL-1/TGF-β signaling pathway tailors behavioral and molecular host responses to a variety of bacteria in Caenorhabditis elegans
Source: eLife. 2023 Sep 26;12:e75831. doi: 10.7554/eLife.75831 (PMC10567113; doi:10.7554/eLife.75831)
Supplement: Supplementary file 1. [file elife-75831-supp1.docx]

Summary of survival assay results reported in Figure 1

| **Means and Medians for Survival Time** | | | | | | | **Pairwise comparisons** | |
| --- | --- | --- | --- | --- | --- | --- | --- | --- |
| Worm Strain | Bacteria | Mean | | | | | *E. coli* OP50 | |
|  |  | Estimate | Std. Error | n | 95% Confidence Interval | | Log Rank (Mantel-Cox) | |
|  |  |  |  |  | Lower Bound | Upper Bound | Chi-Square | Sig. |
| WT | *E. coli* OP50 | 14.98 | 0.35 | 102 | 14.29 | 15.67 |  |  |
| *dbl-1(-)* | *E. coli* OP50 | 14.36 | 0.36 | 104 | 13.64 | 15.17 | 0.909 | 0.34 |
|  |  |  |  |  |  |  |  |  |
| WT | *E. coli* OP50 | 12.30 | 0.37 | 110 | 11.57 | 13.03 | – | – |
|  | *E. cloacae* | 16.22 | 0.39 | 104 | 15.46 | 16.98 | 46.694 | 0.000 |
| *dbl-1(-)* | *E. coli* OP50 | 11.87 | 0.38 | 111 | 11.13 | 12.62 | – | – |
|  | *E. cloacae* | 11.64 | 0.33 | 105 | 10.99 | 12.29 | 1.227 | 0.268 |
|  |  |  |  |  |  |  |  |  |
| WT | *E. coli* OP50 | 12.30 | 0.37 | 110 | 11.57 | 13.03 | – | – |
|  | *K. oxytoca* | 14.34 | 0.31 | 120 | 13.74 | 14.94 | 13.469 | 0.000 |
| *dbl-1(-)* | *E. coli* OP50 | 11.87 | 0.38 | 111 | 11.13 | 12.62 | – | – |
|  | *K. oxytoca* | 12.55 | 0.32 | 108 | 11.93 | 13.16 | 0.067 | 0.796 |
|  |  |  |  |  |  |  |  |  |
| WT | *E. coli* OP50 | 15.19 | 0.23 | 111 | 14.73 | 15.65 | – | – |
|  | *S. marcescens* | 15.09 | 0.28 | 107 | 14.55 | 15.64 | 0.028 | 0.867 |
| *dbl-1(-)* | *E. coli* OP50 | 14.58 | 0.22 | 97 | 14.15 | 15.00 | – | – |
|  | *S. marcescens* | 8.56 | 0.25 | 111 | 8.08 | 9.04 | 160.72 | 0.000 |
|  |  |  |  |  |  |  |  |  |
| WT | *E. coli* OP50 | 14.06 | 0.24 | 95 | 13.59 | 14.53 | – | – |
|  | *B. megaterium* | 16.54 | 0.20 | 84 | 16.13 | 16.94 | 38.335 | 0.000 |
| *dbl-1(-)* | *E. coli* OP50 | 13.99 | 0.22 | 84 | 13.56 | 14.41 | – | – |
|  | *B. megaterium* | 15.25 | 0.22 | 84 | 14.82 | 15.68 | 17.737 | 0.000 |
|  |  |  |  |  |  |  |  |  |
| WT | *E. coli* OP50 | 13.32 | 0.24 | 121 | 12.86 | 13.79 | – | – |
|  | *E. faecalis* | 17.47 | 0.32 | 129 | 16.83 | 18.10 | 94.972 | 0.000 |
| *dbl-1(-)* | *E. coli* OP50 | 13.58 | 0.23 | 122 | 13.13 | 14.04 | – | – |
|  | *E. faecalis* | 14.97 | 0.33 | 126 | 14.32 | 15.62 | 31.621 | 0.000 |

| WT | *E. coli* OP50 | 13.20 | 0.22 | 123 | 12.78 | 13.63 | – | – |
| --- | --- | --- | --- | --- | --- | --- | --- | --- |
|  | *S. epidermidis* | 15.97 | 0.34 | 104 | 15.30 | 16.64 | 58.699 | 0.000 |
| *dbl-1(-)* | *E. coli* OP50 | 13.72 | 0.28 | 129 | 13.17 | 14.27 | – | – |
|  | *S. epidermidis* | 11.88 | 0.35 | 138 | 11.20 | 12.55 | 9.264 | 0.002 |
