# Supplementary material for: The DBL-1/TGF-β signaling pathway tailors behavioral and molecular host responses to a variety of bacteria in Caenorhabditis elegans
Source: eLife. 2023 Sep 26;12:e75831. doi: 10.7554/eLife.75831 (PMC10567113; doi:10.7554/eLife.75831)
Supplement: Supplementary file 3. [file elife-75831-supp3.docx]

List of strains

Strains used include:

| **Strain** | **Genotype** | **RRID** |
| --- | --- | --- |
| N2 | Wild type |  |
| NU3 | *dbl-1(nk3)* V (referred to as *dbl-1(-)* in this work) | WBStrain00029114 |
| CB502 | *sma-2(e502)* III (referred to as *sma-2(-)* in this work) | WBStrain00004174 |
| CB491 | *sma-3(e491)* III (referred to as *sma-3(-)* in this work) | WBStrain00004173 |
| DR1369 | *sma-4(e729)* III (referred to as *sma-4(-)* DR in this work) | WBStrain00006371 |
| LW5558 | *sma-4(jj278)* III (referred to as *sma-4(-)* LW in this work) | WBStrain00026352 |
| LW2436 | *jjIs2277[pCXT51(5*RLR::pes-10p(deleted)::GFP) + LiuFD61(mec-7p::RFP)]* I or IV (RAD-SMAD) | WBStrain00026351 |
| CB6710 | *eEx650[ilys-3p::GFP + unc-119(+)]* | WBStrain00004705 |
| CF3556 | *agIs6[dod-24p::GFP]* | WBStrain00004921 |
| SAL139 | *pha-1(e2123)* III; *denEx17[dod-22::GFP + unc-119(+)]* | WBStrain00033894 |
| SAL143 | *pha-1(e2123)* III; *denEx21[*F55G11.7*::GFP + unc-119(+)]* | WBStrain00033896 |
| SAL148 | *pha-1(e2123)* III; *denEx26[irg-4::GFP + unc-119(+)]* | WBStrain00033899 |

Strains created for this work (available upon request) include:

| **Strain** | **Genotype** |
| --- | --- |
| TLG803 | *dbl-1(nk3)* V; *agIs6[dod-24p::GFP]* |
| TLG804 | *dbl-1(nk3)* V; *eEx650[ilys-3p::GFP + unc-119(+)]* |
| TLG805 | *dbl-1(nk3)* V; *denEx26[irg-4::GFP + unc-119(+)]* |
| TLG806 | *dbl-1(nk3)* V; *denEx21[*F55G11.7*::GFP + unc-119(+)]* |
| TLG807 | *dbl-1(nk3)* V; *denEx17[dod-22::GFP + unc-119(+)]* |
| TLG810 | *jjIs2277[pCXT51(5*RLR::pes-10p(deleted)::GFP) + LiuFD61(mec-7p::RFP)]* I or IV; *dbl-1(nk3)* V |
