# Supplementary material for: The DBL-1/TGF-β signaling pathway tailors behavioral and molecular host responses to a variety of bacteria in Caenorhabditis elegans
Source: eLife. 2023 Sep 26;12:e75831. doi: 10.7554/eLife.75831 (PMC10567113; doi:10.7554/eLife.75831)
Supplement: Supplementary file 4. [file elife-75831-supp4.docx]

List of primers for qRT-PCR

| **Target gene** | **Forward primer (5'->3')** | **Reverse primer (5'->3')** |
| --- | --- | --- |
| *sma-2* | TCCACCAGGAGTTCCAACAT | ACCTGTTCTCCGACTCTTGT |
| *sma-3* | GAGAACACACGGATGCATATTGG | ACTGTGCGGTGGTATTCGG |
| *sma-4* | GATGCTCCGACGTTCTCGAT | CGCATCCTGTCAACTCCACT |
| *act-1* | GCCGGAATCCACGAGACTTC | TCTGGTGGGGCGATGATCTT |
